# The benthic sea-silk-thread displacement of a sessile bivalve, *Pinctada imbricata radiata* (Leach, 1819) in the Arabian-Persian Gulf

**DOI:** 10.1371/journal.pone.0215865

**Published:** 2019-05-01

**Authors:** Bruno Welter Giraldes, Alexandra Leitão, David Smyth

**Affiliations:** 1 Environmental Science Center (ESC), Qatar University (QA), Doha, Qatar; 2 School of Ocean Science Bangor University Menai Bridge, Wales, United Kingdom; University of California, UNITED STATES

## Abstract

A number of molluscs within the Class Bivalvia are defined by their ability to secrete fine silk like threads known as byssus which are used to anchor themselves to solid substrates. With relatively few exceptions the majority of these species remain in a sedentary state throughout their life attached via their byssal threads. However, observations of adult *Pinctada imbricata radiata* pearl oysters made during this study revealed this species’ ability to implement active movement. Byssal threads were secreted in a sequence of attachment and detachment phases, which resulted in the active displacement of the oyster. The oyster was observed, in the laboratory over a 9 day period, travelling a distance of 28cm in a horizontal path. After horizontal displacement, a vertical climbing phase was observed until the oyster reached the water surface at which point the byssus was discarded and the animal dropped, drifting in accordance with water current intensity. It is possible that these adaptations of byssal use are a result of environmentally induced evolutionary change within *P*. *i*. *radiata*.

## Introduction

The Arabian-Persian Gulf is renowned globally as one of the most challenging ecological marine environments due it arid conditions [1,2]. Annual sea temperature variations range from 14–36°C with salinities averaging >39 psu and in some coastal regions >60 psu [1–3]. As a result of these harsh environmental conditions numerous unique ecosystems exist such as; the biogenic reef structures created by the pearl oyster *Pinctada imbricata radiata* (Leach, 1814) [4,5]. These reefs once occurred on an immense scale. Pliny the elder in 38 AD estimated that the oyster beds of the western Gulf covered an area of >1050 Km stretching from Sharjan (in the United Arab Emirates) to Qatif (in Saudi Arabia) [5,6]. However recent research has shown that overexploitation and environmental stressors have resulted in a considerable reduction of the ecotype throughout the region [4].

The pearl oyster *P*. *i*. *radiata* belongs to the family Pteriidae and as a byssal attached bivalve is not considered a “true” oyster by malacologists [7–9]. During the pediveliger ontogeny (after metamorphosis) the settlement process varies within different bivalve groups. Byssal attached species retain the byssus glands producing byssum threads in their adult stages [10–13]. Settlement in bivalves which have motile seed, often display a multi stage event involving; (a) pedal walking, (b) secretion of byssus threads to form temporary or long term attachments and (c) drift dispersal initiated by the secretion of fine byssus that allows drifting [14,15]. The lottery of settlement [16] and the availability of suitable attachment substrates influence many of the processes during this phase of the life cycle. The capacity to secrete byssal (sea silk threads) in the Pteriidae pearl oysters [17] maximises settlement success rates as it allows attachment to a variety of substrates. The fixation or attachment stage observed in species from the genus *Pinctada*, is relatively unique amongst oysters as the functional byssal gland remains active throughout the life cycle [17] and similar to other byssaly attached species the byssum production and attachment can be used for short movements [18]. With the production of byssus governed by several parameters including environmental pressures such as hydrodynamics, salinity, water temperature and pH [18–21].

This research documents a unique observation in adult *P*. *i*. *radiata* whereby byssal threads are used as a means of active transport and not only as an attachment material. Documented observations explain how the byssus can be secreted, used and discarded in a successive action to actively drag the bivalve horizontally over substrate and climb vertical structures.

## Material and methods

*P*. *i*. *radiata* samples were collected by divers during September 2018 from an inshore site close to the coastal port of Semaisma on the eastern coast of Qatar. The oysters were located on a sand/shell substrate at a depth of 5m close to sea grass beds in a water temperature of 32 ^o^C. The specimens were transported from the survey site to the laboratory using an *in-situ* water sample. There is no specific permission required to collect oysters in this site and this study does not involve endangered or protected species.

**Fig 1:**
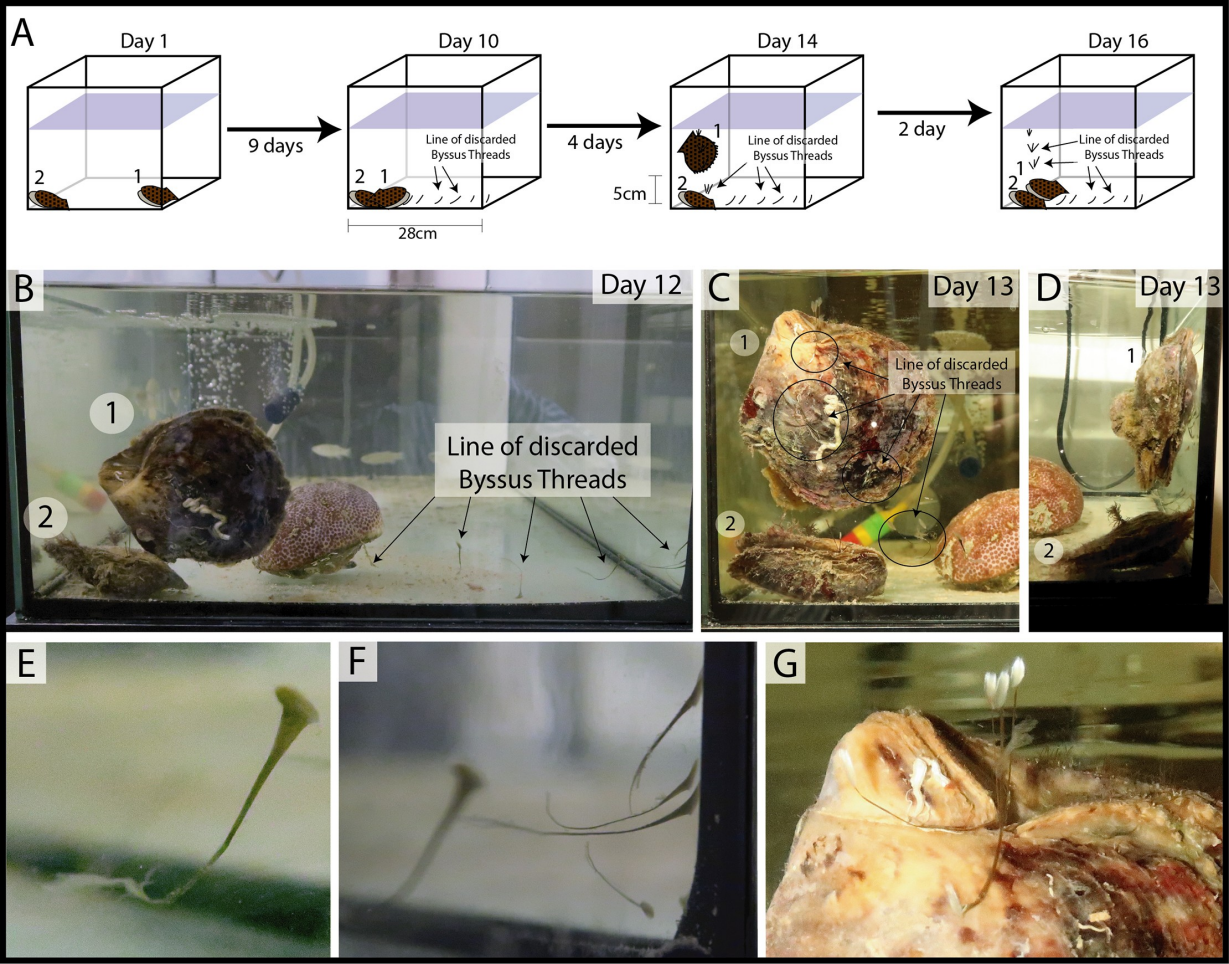
Illustration of sea-silk-thread displacement. (A) The sequence of displacement day 1 to day 16, highlighting the line of the discarded byssum threads of specimen 1; (B) the 28cm displaced by specimen 1 and the line of the discarded byssum threads; the 5 cm climbed (C) in a frontal view with the line of the discarded three-byssum threads and (D) the lateral view; (E,F) details of the discarded byssum threads; (G) details of the three-byssum threads attached in the vertical displacement.

On return to the laboratory, two large individuals (7cm) were placed in a 10lt aerated aquarium containing water from the sample site. The oysters were positioned 28 cm apart independently of each other at the lateral end of the aquarium (Fig 1A). Observations were carried out on a daily basis over a 16-day period with each observation session recorded photographically.

A descriptive account of *in-situ* information is also presented. Specimens were photographed *in-situ* and in the laboratory using an underwater camera (Canon Mark-ii and Fantasea housing FG7X-II).

## Results

Daily Laboratory observations of the Semaisma samples began 24hrs after the oysters had been positioned in the aquarium. The first observational session recorded the visible displacement of one specimen moving towards the other. It was noted that the oyster was secreting a single byssal thread and contracting the muscle in the foot to drag itself along the aquarium. The byssal thread was fixed to the lateral side of the aquarium and discarded after movement had taken place, directly after this event another thread was secreted and attached to repeat a further displacement phase. A recognized sequence of byssal secretion, attachment, drag and discard was observed throughout the sessions. The oyster discarded a linear sequence of horizontally attached byssal threads which followed the path of movement along the aquarium glass (Fig 1A, 1B, 1E and 1F). A total of 28cm was travelled in 9 days (Fig 1A) (S1 Data). The oyster displayed a recognized displacement sequence of secretion, attachment and discard in a determined path of direction (Fig 1E and 1F).

On reaching the second specimen the oyster was observed undertaking a vertical climb of the aquarium wall (Fig 1A, 1C, 1D and 1G). It was noted that the oyster secreted three byssal threads during this vertical movement whereas one byssus was only used in horizontal locomotion (Fig 1G). Once the oyster was positioned vertically it moved 5 cm in 4 days (Fig 1A) (S1 Data) leaving a sequential track-line of discarded three-byssus-treads (Fig 1A and 1C). The oyster was recorded reaching the water surface on the second day, at which point all byssal threads were discarded. On detachment from the byssus the oyster dropped to the aquarium floor drifting in the water current a few centimetres away from the vertical substrate (Fig 1A; day 16). The oyster was then observed replicating the previous climbing actions.

**Fig 2:**
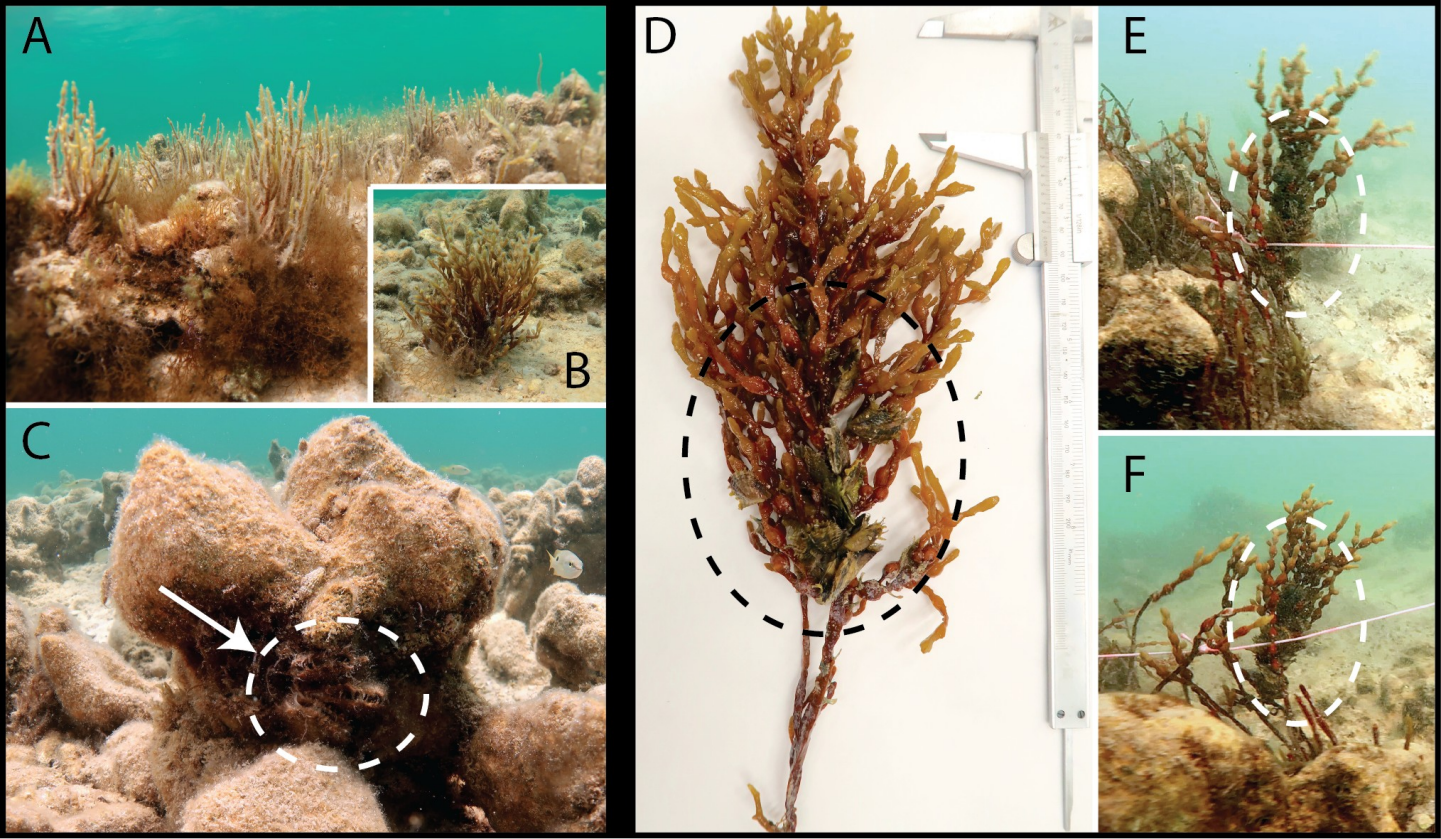
*In-situ* imagery of two locomotive phases observed at a fixed site in Um-Bab, Qatar. First phase (A-C) specimens remain clumped and attached by byssal threads within rock crevices. Second phase (D-F) with the majority of specimens relocated to the fronds of red algae.

In a fixed sample site *in-situ* (Um-Bab), oysters were observed climbing all vertical substrate, including the branches of red algae. Initially the specimens were observed attached in clumps within crevices to among rocks (Fig 2A–2C) and not pro-actively climbing the surrounding red algae. Further observations of the oyster assemblage recorded the clump attached in the upper fronds of the red algae (Fig 2D–2F) the highest vertical *in-situ* topographical feature. The discarded lines of byssus threads in both the basal fronds and on the surface of the rock crevices were clearly visible and marked a direct path of travel.

## Discussion

The basic biology, anatomy and attachment-displacement strategies of bivalves, can be directly related to their modes of life [9]. The mussel families Pinnidae, Mytilidae and Dreissenidae [12] and the false oysters Pteriidae [20] use byssal threads and are considered as byssaly attached species [9]. The observed active displacement of adult *P*. *i*. *radiata* (Pteriidae) using byssus identifies the species within the recognized locomotion-attachment group of the seventh ecological category of bivalves [9] and is similar to other byssaly attached species which present benthic movement during adult stages [18]. However, the locomotive behaviour observed by *P*. *radiata* during this study can be considered unique as it represents the fastest benthic displacement for a byssaly attached species within the group. Comparable studies have recorded bivalve displacement at maximum speeds of < 25cm in one month therefore 28 cm in 9 days represents a significant increase in maximum locomotive speed [18]. This proactive benthic displacement capacity by a byssally attached species has been undocumented until now.

This study adds a new factor in regards to the displacement mechanisms for oyster species within the genus *Pinctada*. A genus which contains 19 species distributed worldwide, several of which have important economic and environmental significance [22]. Displacement capacity has been described previously for some species of this genus in relation to substrate selection and habitual positioning to maximize resources [17,21,23]. However, the description of locomotion recorded during this research has been unknown and the rationale and associations behind the behaviour and the possible implications remains clandestine. The study raises the question if the byssal thread facilitated movement observed in *Pinctada i radiata* is an isolated evolutionary behaviour or is it a phylogenetic trait within the *Pinctada* species. It is probable that the foot is also connected to the observed movements as the threads are produced by the byssal gland located in the foot [24] however only a few groups retain a functional byssus when adult [23]. The adaptation in the use of byssal threads documented in *P*. *i radiata* may have applications in the wild, possibly as an escape strategy from disagreeable environmental conditions or as a means of re-attachment to an oyster reef matrix after dislodgment.

The study species *Pinctada imbricata radiata* is a recognized bioengineering ecosystem-builder responsible for the formation of numerous seascapes within the semi-enclosed region of the western Arabian-Persian Gulf [4,5]. A marine region with naturally occurring extreme environmental conditions [1–3] which can be dated from the mid-Holocene [25]. The pearl oyster endures a considerable suit of natural and anthropogenic stressors within the Gulf region [3–6,26,27]. Challenging situations in nature often force an r-selected species such as *P*. *i*. *radiata* into a situation where evolutionary adaptation is necessary for survival [28,29]. Indeed, several studies have reported on the impact that environmental parameters can have on the quality and number of byssal threads produced with correlations shown between abiotic factors, byssal attachment and overall health [19–21,28,30]. The documented incidents of active displacement in bivalve species have generally been in response to predators or unfavourable environmental conditions [31,32]. Therefore, the sea-silk-thread locomotion observed in this study may represent an evolutionary adaptation within the species. The behaviour offers the bivalve a means of escaping hazardous conditions or compromised habitual niches. The vertical climbing and byssus detachment observed in the laboratory has practical applications for the oyster in the wild in regards to a current induced drift escape mechanism. In the wild the oyster has been observed *in-situ* climbing above the sea floor using rocks, algal stipe and fronds (Fig 2) to gain height. It is possible that with this height advantage *P*. *radiata* could discard its byssus from the algae and utilize tidal velocities to avoid predators and seasonal temperature extremes, which are common occurrences in the region [27,33].

In conclusion, the description of benthic displacement by adults of *P*. *i*. *radiata* is of significant importance in understanding the functional responses of this species to abiotic and biotic drivers within the Gulf region. The documentation of byssal induced locomotion will also assist fishery and habitat managers who intend to use the ecosystem services provided by this oyster in assisting the restoration of environmentally compromised sites.

## Supporting information

S1 Data Measurements of the observed behaviour highlighting the displacement (cm) per day and the direction (horizontal or vertical) and the number of byssum thread used in each movement. (XLSX)
